# Investigation of optimal weight gain during pregnancy for Japanese Women

**DOI:** 10.1038/s41598-017-02863-1

**Published:** 2017-05-31

**Authors:** Kyoko Nomura, Michiko Kido, Ayumi Tanabe, Kengo Nagashima, Shinichi Takenoshita, Kazumichi Ando

**Affiliations:** 1 Department of Hygiene and Public Health, School of Medicine, Teikyo University, Tokyo, 173-0003 Japan; 2 Department of Obstetrics and Gynecology, Japanese Red Cross Medical Center, Tokyo, 150-8935 Japan; 3 Department of Preventive Medicine and Public Health, Keio University School of Medicine, Tokyo, 160-8582 Japan; 4 Department of Global Clinical Research, Graduate School of Medicine, Chiba University, Chiba, 260-8677 Japan

## Abstract

This study aims to compare the US Institute of Medicine (IOM) and Japanese guidelines proposed by the Ministry and the Japan Society for the Study of Obesity on gestational weight gain (GWG), and to explore the optimal GWG range in Japanese women. We investigated 8,152 Japanese women who had full-term singleton babies between 2010 and 2013 at a single center in Tokyo. Logistic regression models showed that GWG below the recommendation of the IOM and Japanese guidelines was similarly associated with an increased risk of light-for-date (LFD), whereas GWG above these guidelines was similarly associated with an increased risk of heavy-for-date (HFD) in pre-pregnancy body mass index categories of underweight (<18.5 kg/m^2^, n = 1559), normal-weight (18.5–24.9 kg/m^2^, n = 4998), overweight (25.0–29.9 kg/m^2^, n = 270), and obese (30 ≤ kg/m^2^, n = 60). The receiver-operating characteristic curve demonstrated that the optimal cutoffs for LFD and HFD were 9.7 and 10.4 kg, respectively in normal-weight mothers. The IOM and Japanese guidelines identified the risk of LFD or HFD equally well. The optimal GWG range in normal-weight women observed in this study was more close to Japanese guideline (i.e., 7–12 kg) compared to the IOM guideline (i.e., 11.5–16 kg).

## Introduction

Maternal weight prior to and during pregnancy affects the health of mothers and children. Underweight women tend to have low birth weight babies compared to obese woman while obese women tend to have large babies compared to underweight women. A low birth weight infant, defined as a birth weight <2,500 g at delivery, is associated with an increased risk of poor health in adulthood, including an increased risk of cardiovascular disease and diabetes^1^. Macrosomia, defined as a birth weight greater than 4,000 g at delivery is an independent risk factor for complications at delivery, i.e., critical hemorrhage, immediate adverse health effects, i.e., gestational diabetes and pregnancy-induced hypertension, and an increased risk of obesity in childhood and young adulthood^2^.

The US Institute of Medicine (IOM) published gestational weight gain guidelines for women in Western countries based on pre-pregnancy body mass index (BMI). In those guidelines, women were categorized as underweight (BMI < 18.5 kg/m^2^; recommended gestational weight gain [GWG], 12.5–18 kg), normal-weight (BMI 18.5–24.9 kg/m^2^; 11.5–16 kg), overweight (BMI 25–29.9 kg/m^2^; 7–11.5 kg), and obese (BMI ≥ 30; 5–9 kg). These guidelines were intended to balance the risks of having large-for-gestational-age infants, small-for-gestational-age infants, preterm births, and postpartum weight retention in obese women^3^. The IOM guidelines are useful for Western countries, where overweight and obese women are more prevalent; however, Asian women are smaller, and their gestational weight gain is different from that of women of other ethnicities. In 2016, the Japanese Society of Obstetrics and Gynecology Successive Pregnancy Birth Registry System^4^ performed a large-scale study of 97,157 pregnant Japanese women, which showed that the average weight gain during pregnancy was 10.3 kg in underweight, 10.1 kg in normal-weight, 7.9 kg in overweight, and 5.5 kg in obese women. Notably, all such weight gains were much lower than the ranges in the IOM guidelines.

Public attention has increased for underweight women in Japan since the 2015 national Nutrition Survey^5^ revealed that one-fourth of women of reproductive age were categorized as underweight, indicating that their average energy intake was lower than the dietary reference intake^6^. Moreover, the prevalence of low birth weight babies has increased in Japan from 5.2% in 1980 to 9.6% in 2010. Notably, a low birth weight baby is associated with maternal body weight prior to and during pregnancy. Thus, the appropriate GWG range to prevent a birth of a low birth weight baby requires immediate clarification.

Guidelines detailing appropriate GWG ranges were issued by the Japanese Ministry for Health, Labor, and Welfare (JMHLW)^7^ for underweight (recommended GWG, 9–12 kg) and normal-weight (recommended GWG, 7–12 kg) women, and by the Japanese Society for the Study of Obesity (JSSO) for overweight (recommended GWG ≤ 7 kg) and obese (recommended GWG ≤ 5 kg) women^8^. However, the evidence supporting these recommendations is limited. Hence, the purpose of this study was to assess the accuracy of those guidelines for Japanese women and to determine the optimal GWG based on pre-pregnancy BMI.

## Results

### Baseline characteristics according to a level of pre-pregnancy BMI

Table 1 shows the baseline characteristics for individuals included in this study. The mean maternal age was lowest for underweight mothers and increased with pre-pregnancy BMI (*P* < 0.0001). The mean GWG was 10.5 kg for underweight women, 10.2 kg for normal-weight women, 7.4 kg for overweight women, and 4.6 kg for obese women (*P* < 0.0001). The proportion of cesarean sections also increased with increases in pre-pregnancy BMI (*P* < 0.0001). Smoking rates (*P* = 0.010) and the prevalence of a heavy-for-date (HFD) infant (*P* < 0.0001) were highest among obese mothers, whereas a light-for-date (LFD) infant (*P* < 0.0001) was most prevalent in underweight mothers.

**Table 1:** Baseline characteristics according to a level of pre-pregnancy BMI.

	Underweight	Normal weight	Overweight	Obese	*P*-value
	BMI < 18.5 kg/m^2^	18.5 ≤ BMI < 25 kg/m^2^	25 ≤ BMI < 30 kg/m^2^	30 ≤ BMI kg/m^2^	
	n = 1559	n = 4998	n = 270	n = 60	
	N (%) or Mean ± SD	N (%) or Mean ± SD	N (%) or Mean ± SD	N (%) or Mean ± SD	
Maternal age, years	33.0 ± 4.8	34.3 ± 4.8	35.3 ± 5.1	34.6 ± 4.8	< 0.001
Maternal height, cm	160 ± 5.4	159 ± 5.4	159 ± 5.5	157 ± 8.1	< 0.001
Pre-pregnancy weight, kg	45.2 ± 3.5	52.3 ± 5.0	67.3 ± 5.7	80.2 ± 8.2	< 0.001
Pre-pregnancy BMI, kg/m^2^	17.6 ± 0.8	20.6 ± 1.5	26.7 ± 1.3	32.5 ± 3.0	< 0.001
BMI at delivery, kg/m^2^	21.7 ± 1.4	24.6 ± 1.9	29.7 ± 2.0	34.4 ± 3.3	< 0.001
Gestational weight Gain, kg	10.5 ± 3.2	10.2 ± 3.3	7.4 ± 4.4	4.6 ± 4.3	< 0.001
Gestational Week	39.2 ± 1.1	39.3 ± 1.1	39.3 ± 1.1	39.4 ± 1.0	0.033
Parity^a^					0.007
Nulliparity	996 (68.4)	3001 (64.1)	150 (59.5)	36 (64.3)	
Multiparity	461 (31.6)	1678 (35.9)	102 (40.5)	20 (35.7)	
Delivery Method					<0.001
Cesarean section	168 (10.8)	914 (18.3)	83 (30.7)	16 (26.7)	
Vaginal Delivery	1391 (89.2)	4084 (81.7)	187 (69.3)	44 (73.3)	
Smoking^a^					0.010
Current smoking	36 (2.3)	72 (1.4)	4 (1.5)	2 (3.3)	
Past smoking	200 (12.8)	605 (12.1)	47 (17.4)	12 (20.0)	
Never	1323 (84.9)	4318 (86.5)	219 (81.1)	46 (76.7)	
Drinking^a^					0.064
Current drinking	42 (2.7)	102 (2.1)	8 (3.0)	0 (0.0)	
Past drinking	356 (22.9)	1197 (24.0)	61 (22.6)	6 (10.0)	
Neve	1157 (74.4)	3684 (73.9)	201 (74.4)	54 (90.0)	
Light for date	175 (11.2)	371 (7.4)	21 (7.8)	2 (3.3)	<0.001
Heavy for date	109 (7.0)	543 (10.7)	47 (17.4)	13 (21.7)	<0.001

Based on a chi-square test or a Fisher Exact test for categorical variables and an analysis of variance for continuous variables.

^a^Summation does not reach total number due to missing values.

### Odds risks for a LFD or HFD infant among underweight, normal-weight, overweight and obese mothers

The odds risks for LFD or HFD are shown in Table 2 according to pre-pregnancy BMI categories. Among underweight mothers (n = 1559), 32% (n = 495) experienced weight gains below the JMHLW recommendations and 27% (n = 423) experienced above the guideline. After adjusting for smoking selected by stepwise model selection, weight gain below the IOM guidelines of 12.5 kg [OR 1.77, 95% Confidence Interval (CI): 1.15–2.72] or the JMHLW guideline of 9 kg (OR 1.74, 95% CI: 1.23–2.48) was significantly associated with an increased risk of LFD. Weight gain above the IOM guideline of 18 kg (OR 1.91, 95% CI: 1.15–3.17) or the JMHLW guideline of 12 kg (OR 2.23, 95% CI: 1.40–3.56) was significantly associated with an increased risk of HFD adjusting for nulliparity that was selected by stepwise model selection.

**Table 2:** Odds risks for a Light-for-date (LFD) or Heavy-for-date (HFD) infant according to pre-pregnancy BMI categories.

		Variable (no. of events)	Univariate			Stepwise Multivariate Mode	
			N (%)	OR (95% CI)	p-value (Type III)	OR (95% CI)	p-value (Type III)
pre-pregnancy BMI < 18.5 kg/m^2^ (n = 1559)							
IOM	<12.5 kg (N = 1184)	LFD (175)	148 (12.5)	1.71 (1.11–2.62)	0.050	1.77 (1.15–2.72)^a^	0.036
	12.5–18 kg (N = 350)		27 (7.7)	1.00 (reference)		1.00 (reference)	
	>18 kg (N = 25)		0 (0.0)	NA		NA	
JMHLW	<9 kg (N = 495)		61 (12.3)	1.71 (1.20–2.43)	<0.000	1.74 (1.23–2.48)^a^	<0.000
	9–12 kg (N = 641)		55 (8.6)	1.00 (reference)		1.00 (reference)	
	>12 kg (N = 423)		19 (4.5)	0.68 (0.43–1.06)		0.65 (0.41–1.02)^a^	
IOM	<12.5 kg (N = 1184)	HFD (109)	62 (5.2)	0.43 (0.28–0.65)	<0.000	0.45 (0.29–0.70)^b^	<0.000
	12.5–18 kg (N = 350)		40 (11.4)	1.00 (reference)		1.00 (reference)	
	>18 kg (N = 25)		7 (28.0)	3.01 (1.19–7.66)		1.91 (1.15–3.17)^b^	
JMHLW	<9 kg (N = 495)		22 (4.4)	0.78 (0.45–1.35)	<0.000	0.84 (0.48–1.48)^b^	<0.001
	9–12 kg (N = 641)		36 (5.6)	1.00 (reference)		1.00 (reference)	
	>12 kg (N = 423)		51 (12.1)	2.30 (1.48–3.60)		2.23 (1.40–3.56)^b^	
pre-pregnancy BMI 18.5–24.9 kg/m^2^ (n = 4998)							
IOM	<11.5 kg (N = 3331)	LFD (371)	286 (8.6)	1.68 (1.29–2.17)	<0.000	1.73 (1.33–2.25)^c^	<0.000
	11.5–16 kg (N = 1451)		77 (5.3)	1.00 (reference)		1.00 (reference)	
	>16 kg (N = 216)		8 (3.7)	0.69 (0.33–1.44)		0.65 (0.31–1.14)^c^	
JMHLW	<7 kg (N = 691)		86(12.5)	1.75 (1.34–2.28)	<0.000	1.76 (1.35–2.30)^c^	<0.000
	7–12 kg (N = 3028)		227 (7.5)	1.00 (reference)		1.00 (reference)	
	>12 kg (N = 1279)		58 (4.5)	0.59 (0.44–0.79)		0.56 (0.42–0.76)^c^	
IOM	<11.5 kg (N = 3331)	HFD (543)	276 (8.3)	0.53 (0.44–0.65)	<0.000	0.51 (0.43–0.63)^d^	<0.000
	11.5–16 kg (N = 1451)		210 (14.5)	1.00 (reference)		1.00 (reference)	
	>16 kg (N = 216)		48 (22.2)	1.69 (1.19–2.40)		1.77 (1.24–2.51)^d^	
JMHLW	<7 kg (N = 691)		40 (5.8)	0.58 (0.41–0.81)	<0.000	0.57 (0.41–0.80)^d^	<0.00
	7–12 kg (N = 3028)		291 (9.6)	1.00 (reference)		1.00 (reference)	
	>12 kg (N = 1279)		203 (15.9)	1.77 (1.46–2.15)		1.83 (1.51–2.22)^d^	
pre-pregnancy BMI 25–29.9 kg/m^2^ (n = 270)							
IOM	<7 kg (N = 119)	LFD(21)	15 (12.6)	2.48 (0.92–6.63)	0.071	3.37 (1.20–9.47)^e^	0.021
	7–11.5 kg (N = 109)		6 (5.5)	1.00 (reference)		1.00 (reference)	
	>11.5 kg (N = 42)		0 (0.0)	NA		NA	
JSSO	≤7 kg (N = 124)		15 (12.1)	3.21 (1.21–8.55)	0.020	4.00 (1.45–11.00)^e^	0.007
	>7 kg (N = 146)		6 (4.1)	1.00 (reference)		1.00 (reference)	
IOM	<7 kg (N = 119)	HFD(47)	11 (9.2)	0.38 (0.18–0.82)	0.004	Covariates not selected	
	7–11.5 kg (N = 109)		23 (21.1)	1.00 (reference)			
	>11.5 kg (N = 42)		13 (31.0)	1.68 (0.75–3.73)			
JSSO	≤7 kg (N = 124)		14 (11.3)	1.00 (reference)	0.016	Covariates not selected	
	>7 kg (N = 146)		33 (22.6)	2.30 (1.17–4.52)			
pre-pregnancy BMI 30 ≤ kg/m^2^ (n = 60)							
IOM	<5.0 kg (N = 29)	LFD(2)	1 (3.5)	0.64 (0.04–10.94)	0.954	Covariates not selected	
	5.0–9.0 kg (N = 19)		1 (5.3)	1.00 (reference)			
	>9.0 kg (N = 12)		0 (0.0)	NA			
JSSO	≤5 kg (N = 32)		1 (3.1)	0.87 (0.05–14.60)	0.924	Covariates not selected	
	>5 kg (N = 28)		1 (3.6)	1.00 (reference)			
IOM	<5.0 kg (N = 29)	HFD (13)	5 (17.2)	0.78 (0.18–3.38)	0.532	Covariates not selected	
	5.0–9.0 kg (N = 19)		4 (21.1)	1.00 (reference)			
	>9.0 kg (N = 12)		4 (33.3)	1.88 (0.37–9.57)			
JSSO	≤5 kg (N = 32)		6 (18.8)	1.00 (reference)	0.559	Covariates not selected	
	>5 kg (N = 28)		7 (25.0)	1.44 (0.42–4.96)			

^a^Adjusting for smoking in the estimation of the risk for LFD.

^b^Adjusting for nulliparity in the estimation of the risk for HFD.

^c^Adjusting for age, cesarean section, and smoking in the estimation of the risk for LFD.

^d^Adjusting for age and cesarean section in the estimation of the risk for HFD.

^e^Adjusting for cesarean section in the estimation of the risk for LFD.

Among normal-weight mothers (n = 4998), 14% (n = 691) experienced GWG below the JMHLW recommendations and 26% (n = 1279) experienced above the guideline. After adjusting for age, cesarean section, and smoking selected by a stepwise model, weight gain below the IOM guidelines of 11.5 kg [OR 1.73, 95% Confidence Interval (CI): 1.33–2.25] or the JMHLW guideline of 7 kg (OR 1.76, 95% CI: 1.35–2.30) was significantly associated with an increased risk of LFD. Weight gain above the IOM guideline of 16 kg (OR 1.77, 95% CI: 1.24–2.51) or the JMHLW guideline of 12 kg (OR 1.83, 95% CI: 1.51–2.22) was significantly associated with an increased risk of HFD adjusting for age and cesarean section selected by a stepwise model selection method.

Among overweight mothers (n = 270), 46% (n = 124) experienced GWG below the JSSO recommendations and 54% (n = 146) experienced above the guideline. After adjusting for cesarean section selected by a stepwise model, weight gain below the IOM guidelines of 7 kg (OR 3.37, 95% CI: 1.20–9.47] or the JSSO guideline of 7 kg (OR 4.00, 95% CI: 1.45–11.00) was significantly associated with an increased risk of LFD. Stepwise logistic models did not find any covariates in overweight women, and accordingly univariate logistic regression models demonstrated that weight gain above the IOM guideline of 11.5 kg (OR 1.68, 95% CI: 0.75–3.73) or the JSSO guideline of 7 kg (OR 2.30, 95% CI: 1.17–4.52) was significantly associated with an increased risk of HFD.

Among obese mothers (n = 60), 53% (n = 32) experienced GWG below the JSSO recommendations and 47% (n = 28) experienced above the guideline. Stepwise model did not select any covariates for adjustment in obese women, and accordingly univariate logistic regression models demonstrated that weight gain below the IOM guidelines of 5 kg or the JSSO guideline of 5 kg was not significantly associated with an increased risk of LFD. Stepwise logistic models did not find any covariates and accordingly univariate logistic regression models demonstrated that weight gain above the IOM guideline of 5 kg or the JSSO guideline of 5 kg was not significantly associated with an increased risk of HFD.

### Area under the ROC curve (AUC) and sensitivity, specificity at the optimal cutoff point

**Figure 1:**
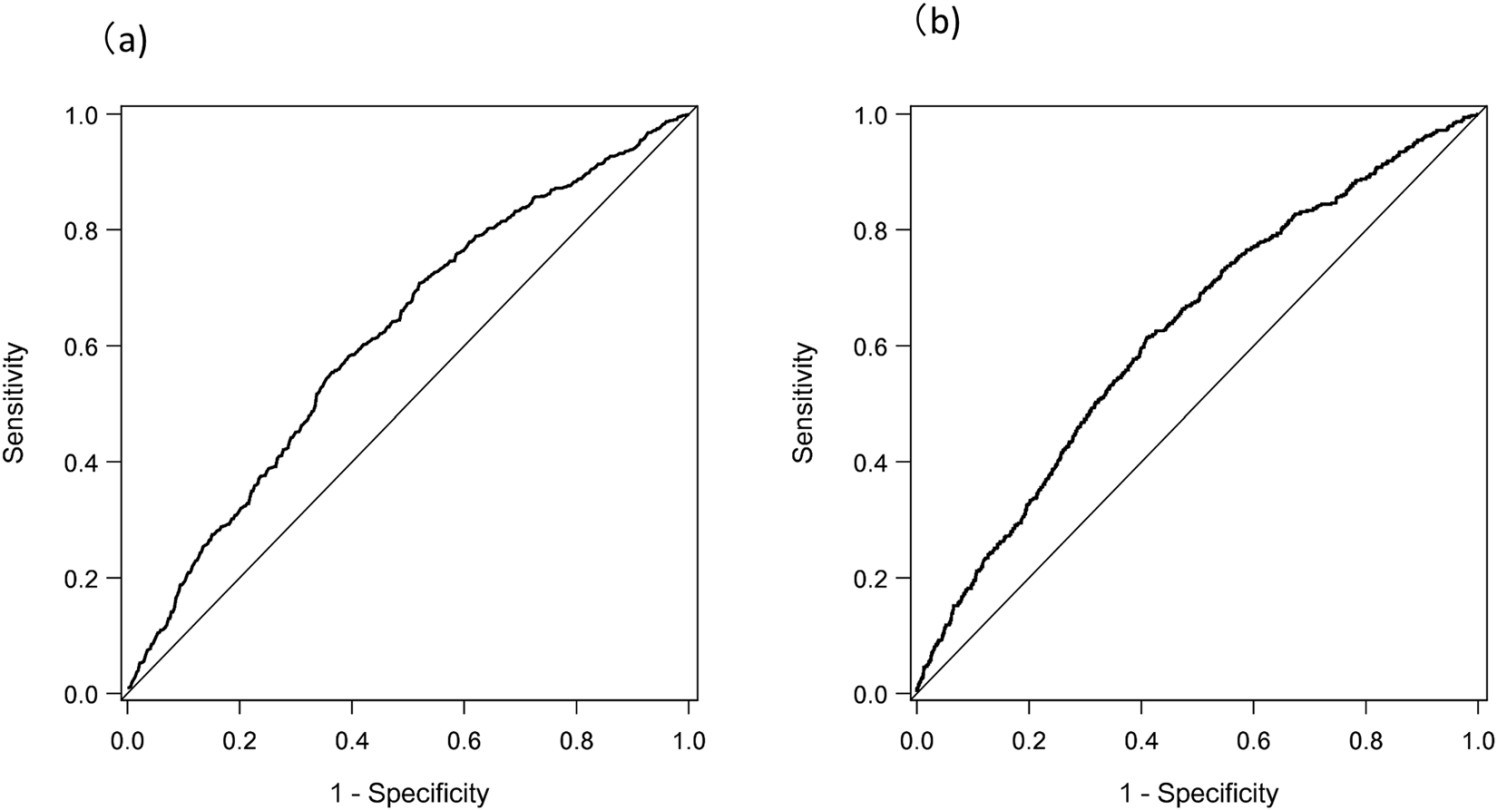
The covariate-adjusted receiver-operating characteristics curve of gestational weight gain for a light-for-date infant (**a**) and for a heavy-for-date infant (**b**). The receiver-operating characteristics curve of gestational weight gain in the risk for a light-for-date infant (**a**) and for a heavy-for-date infant (**b**) was drawn based on Pepe & Longton method. Adjusted avariables selected at a stepwise multivariable logistic analysis included age, cesarean section, and smoking for a light-for-date infant (**a**) and age and cesarean section for a heavy-for-date infnat (**b**).

Table 3 shows area under the ROC curve and sensitivity, specificity at the optimal cutoff point in normal-weight mothers (n = 4998). The area under the adjusted-covariates receiver-operating characteristic (ROC) curves (AUC) was between 0.61 for LFD (Fig. 1a) and 0.62, for HFD (Fig. 1b), which is considered acceptable. The adjusted model demonstrated that optimal cutoffs for LFD and HFD among normal-weight mothers were 9.7 kg and 10.4 kg, respectively, which were more close to the GWG range supported by the JMHLW (7–12 kg) compared to the GWG suggested by the IOM guideline (i.e., 11.5–17 kg).

**Table 3:** Area under the ROC curve (AUC) and Sensitivity, specificity for at the optimal cutoff point in Normal weight. 18.5 ≤ BMI < 25 kg/m^2^ (n = 4998).

Outcome	Crude				Adjusted			
	GWG cutoff	AUC	Sensitivity	Specificity	GWG cutoff	AUC	Sensitivity	Specificity
LFD^a^	9.70	0.61 (0.58–0.64)	0.63 (0.58–0.67)	0.57 (0.55–0.58)	9.70	0.61 (0.58–0.64)	0.64 (0.58–0.69)	0.55 (0.46–0.60)
HFD^b^	10.9	0.62 (0.59–0.64)	0.57 (0.54–0.62)	0.61 (0.60–0.63)	10.40	0.62 (0.60–0.65)	0.61 (0.55–0.65)	0.60 (0.54–0.62)

^a^Adjusting for age, cesarean section, and smoking.

^b^Adjusting for age and cesarean section.

## Discussion

This study compared the risk of LFD or HFD associated with below or above the GWG recommendation of the IOM or JMHLW guideline in underweight and normal-weight mothers, and the GWG recommendation of the IOM and JSSO guideline in overweight and obese mothers, and also determined the optimal GWG during pregnancy in normal-weight women. The IOM and the two Japanese guidelines similarly discriminated the risks of LFD or HFD in underweight, normal-weight, and overweight mothers, whereas there was no significant findings observed in obese mothers but this may be due to small sample size of this group. In normal-weight women, the GWG range found in this study (9.7–10.4 kg) was close to the current JMHLW recommendation (7–12 kg) compared to the GWG proposed by the IOM guideline (i.e., 11.5–17 kg).

In 1990, the IOM revised the guidelines for weight gain during pregnancy, and recommended optimal weight gain ranges based on pre-pregnancy BMI^3^. Due to the absence of official GWG recommendations in Asian countries, including China^9^ and Taiwan^10^, the IOM guidelines are generally followed. In 2016, large-scale retrospective studies of 97,157 Japanese women^4^ and 48,867 Chinese women^11^ found that the IOM guidelines can be applied to Asian women in that they can statistically assess the risk of delivery complications including LFD or HFD. However, Asian women are smaller and experience lower weight gains; therefore, excessive weight gain may lead to harmful events^12, 13^, including macrosomia, preterm birth, preeclampsia, gestational diabetes, pregnancy-induced hypertension, and short- and long-term postpartum weight retention^14^. Thus, an accurate GWG range should be determined for Asian women.

One study^15^ based on 5,351 singleton pregnancies compared the odds ratio for JMHLW- and IOM-recommended GWG ranges and found that the risk of LFD was increased among underweight (9–12 kg vs. 12.8–18.1 kg, OR 2.53, 95% CI: 1.18–5.40), normal-weight (7–12 kg vs. 11.3–15.9 kg, OR 1.72, 95% CI: 1.33–2.23), and overweight mothers (≤7 kg vs. 6.8–11.3 kg OR 2.62, 95% CI: 1.06–6.44). Those data suggest that the current JMHLW recommendations for GWG can identify health risks in pregnant Japanese women better than the IOM guidelines can^15^.

Although previous studies investigated the accuracy of guidelines by estimating odds ratios, the current study also investigated GWG ranges according to optimal cutoffs using ROC curves to predict LFD or HFD in normal-weight women. Among these individuals, the optimal GWG range was 9.7–10.4 kg, which is included within the JMHLW recommendation of 7–12 kg. This GWG range was also supported by a study of 956 Taiwanese women that found an optimal range of 10–14 kg in normal-weight mothers^10^.

One strength of this study is that the dataset contained smoking information, which was absent in one of the largest epidemiological study in Japan^4^. Smoking is an independent risk factor for low birth weight babies, and it is therefore necessary to include this information especially in the estimation of the risk for LFD. In our study, smoking was selected by a stepwise multivariable model to estimate the risk for LFD in underweight and normal-weight women but overweight or obese groups; this was because the overweight and obese populations were too small for covariate-adjusted analyses. One limitation of this study was that all individuals analyzed were from a single hospital in Tokyo. Therefore, they may have been more affluent, healthy, and educated compared to the general population. Additionally, patients may have had unknown risk factors or complications during pregnancy given that the hospital is an advanced prenatal center. Thus, the applicability of the data may be limited to pregnant women in a large city such as Tokyo. Third, pre-pregnancy weight was measured based on a self-reporting which may cause information bias. In this regard, we performed the additional analyses by using maternal weight measured at 20–25 weeks and confirmed the result was not changed in normal-weight women; due to a large number of missing values on this variable, the analyses were unable to be performed in underweight, overweight, and obese women though. Fourth, we used customized birth weight centile charts that provide gestational week based birth weight according to gender of an infant. However, our dataset did not have the information of infant gender and thus we used an average of birthweight between a girl and a boy, which may induce bias. Fifth, in this study, our dataset does not have the information about diet, physical activity, which may affect weight gain during pregnancy. Sixth, ideal GWG ranges would be based on multiple factors, including parity, postpartum hemorrhage, preterm birth, gestational diabetes, pregnancy-induced hypertension, preeclampsia, and postpartum weight retention^16^. Thus, there might have been unmeasured confounders that should be included in our analyses. Seventhly, adjustments based on ROC curves were limited to normal-weight mothers due to small sample sizes of underweight, overweight and obese women. In spite of large sample size of normal-weight women, the AUC values for both LFD and HFD are slightly low (i.e., 0.61 and 0.62, respectively) compared to a reference of the acceptance (i.e., 0.60–0.80), and therefore there may be limited use in applying ROC curve to estimate GWG range. Finally, a large baby is known to be associated with a family history of diabetes mellitus^12^; however, the current study did not include family histories. Hence, the result of our study requires careful interpretations.

In conclusion, no evidence was found in this study to change the current Ministry-recommended GWG guidelines for mothers with singleton babies regardless of pre-pregnancy BMI levels. This study also found that based on the covariate adjusted ROC curve, GWG of 9.7–10.4 kg in normal-weight mothers is suitable to minimize the risk of LFD or HFD.

## Methods

### Study design and participants

**Figure 2:**
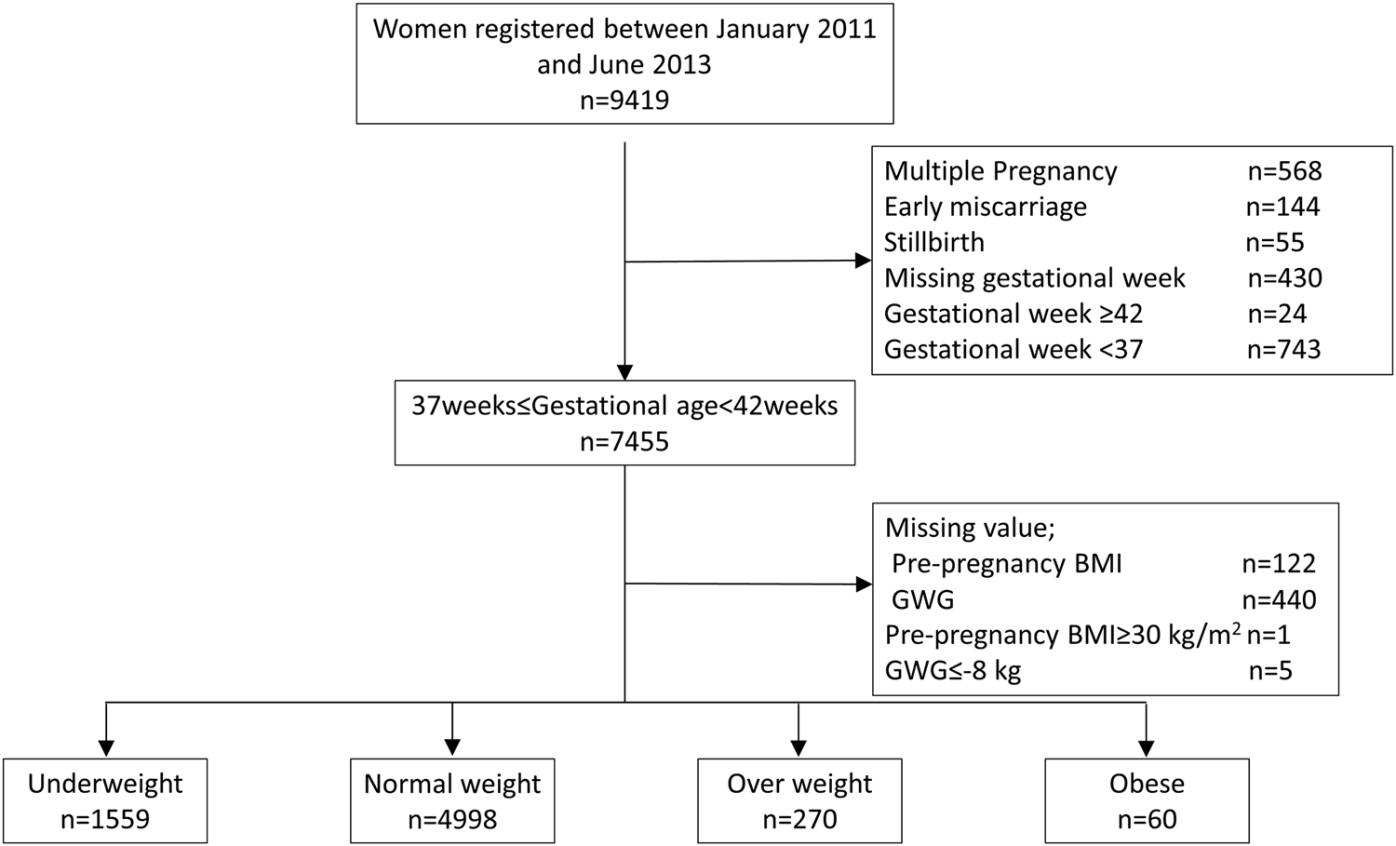
Title and Legend: Flowchart showing total number of participants enrolled and the final number of participants according to pre-pregnancy BMI categories included in the analyses.

This cross-sectional study evaluated consecutive deliveries performed at a single hospital between January 2010 and June 2013. The hospital is a Red Cross medical Center in the Tokyo Metropolitan Area, which experiences the second largest number of deliveries in Tokyo. A total of 9,419 mothers visited and registered at the study site during the 3.5 years, and after excluding individuals for early miscarriages (n = 144), multiple pregnancies (n = 568), and stillbirths (n = 55), the dataset comprised 8,652 individuals who gave birth to singleton babies. This study focused on full-term deliveries, so individuals were also excluded due to preterm births (birth before 37 gestational weeks; n = 743), post-term births (births at 42 weeks or later; n = 24), or unverified gestational week (n = 430). Individuals with missing pre-pregnancy BMI (n = 122) or GWG data (n = 440) were also excluded, as were individuals with gestational weight loss during pregnancy of <8 kg (n = 5), who were considered outliers. Thus, a total of 6,887 individuals were assessed in this study (underweight, n = 1559; normal-weight, n = 4998; overweight, n = 270; and obese, n = 60). All participants provided written informed consent and the study enrollment is shown in Fig. 2. The ethics committee at the Teikyo University School of Medicine, Tokyo, Japan, approved this study (TU-COI 13–1592), confirming that all methods were performed in accordance with the relevant guidelines and regulations.

### Outcome

LFD and HFD were the outcomes considered in this study, which refer to an infant born with a birth weight less than the 10th percentile and more than the 90th percentile for its estimated gestational age, respectively. We used the latest version of customized birth weight centile charts for Japanese issued by Japan Pediatric Society^17^. This new charts provide gestational week based birth weight according to gender of an infant. Because our dataset did not have the information of infant gender, we estimated an average of birthweight between a girl and a boy according to gestational week.

### Pre-pregnancy BMI categories, weight gain during pregnancy, and covariates

Pre-pregnancy weight was obtained by the self-reporting and maternal height was measured at outpatient clinic for the first prenatal visit. Maternal weight at delivery was measured within 2 days after delivery and thus pre-pregnancy BMI and BMI at delivery were calcul

ated as mother’s weight obtained at two different points in time divided by her height squared. Weight gain during pregnancy (i.e., GWG) was calculated by subtracting pre-pregnancy weight from maternal weight at delivery.

Participants were categorized by pre-pregnancy BMI as underweight (<18.5 kg/m^2^), normal-weight (18.5–24.9 kg/m^2^), overweight (25–29.9 kg/m^2^), and obese (≥30 kg/m^2^). Other factors considered were maternal age, parity, gestational week, delivery method, and smoking and drinking habits (current/past/never).

### Statistical analyses

An association between pre-pregnancy BMI and baseline characteristics was assessed using the chi-square or the Fisher’s exact test for categorical variables. Analysis of variance was used to assess continuous variables. Logistic regression analysis was used to estimate the odds ratios and 95% CI for GWG and covariates selected by a stepwise selection method were put into multivariable models. The recommended GWG by the IOM was compared to that by the JMHLW guideline for underweight and normal-weight individuals^7^, and compared to that by the JSSO for overweight and obese individuals^8^. Based on logistic regression models, crude ROC curves and covariate-adjusted ROC curves were used to determine the optimal cutoffs for Youden’s index^18^. CIs for the AUC and covariate-adjusted ROC curves were calculated based on the method of DeLong *et al*.^19^ or Pepe *al*.^20^. AUC values between 0.6 and 0.8 were considered acceptable based on Hosmer and Lemeshow^21^. Due to the limited sample size, we only focused on normal-weight women to estimate GWG with the lower limit which was considered the optimal cut-off for LFD, and the upper limit which was considered the optimal cutoff for HFD.

All data were analyzed using SAS version 9.4 for Windows (Cary, NC, USA) and Stata version 14.2 (Stata Corp., College Station, TX, USA). All CIs were estimated at the 95% level, and *P*-values <0.05 were considered statistically significant.

### Data Availability

The data that support the findings of this study might be available from Japanese Red Cross Medical Center but restrictions apply to the availability of these data based on the ethical committee decision, and so are not be publicly available. Data might be however available from the authors upon reasonable request and with permission of Japanese Red Cross Medical Center.
